# Comparison result of inversion of gravity data of a fault by particle swarm optimization and Levenberg-Marquardt methods

**DOI:** 10.1186/2193-1801-2-462

**Published:** 2013-09-14

**Authors:** Reza Toushmalani

**Affiliations:** Department of Computer, Faculty of engineering, Kangavar Branch, Islamic Azad University, Kangavar, Iran

**Keywords:** Particle swarm optimization, Levenberg-Marquardt method, Inversion, Gravity data, Fault

## Abstract

The purpose of this study was to compare the performance of two methods for gravity inversion of a fault. First method [Particle swarm optimization (PSO)] is a heuristic global optimization method and also an optimization algorithm, which is based on swarm intelligence. It comes from the research on the bird and fish flock movement behavior. Second method [The Levenberg-Marquardt algorithm (LM)] is an approximation to the Newton method used also for training ANNs. In this paper first we discussed the gravity field of a fault, then describes the algorithms of PSO and LM And presents application of Levenberg-Marquardt algorithm, and a particle swarm algorithm in solving inverse problem of a fault. Most importantly the parameters for the algorithms are given for the individual tests. Inverse solution reveals that fault model parameters are agree quite well with the known results. A more agreement has been found between the predicted model anomaly and the observed gravity anomaly in PSO method rather than LM method.

## Introduction

Optimization has been an active area of research for several decades. As many real-world optimization problems become more complex, better optimization algorithms were needed. In all optimization problems the goal is to find the minimum or maximum of the objective function. Thus, unconstrained optimization problems can be formulated as minimization or maximization of D dimensional function:1

where D is the number of parameters to be optimized. Many population based algorithms were proposed for solving unconstrained optimization problems. Genetic algorithms (GA), particle swarm optimization (PSO), are most popular optimization algorithms which employ a population of individuals to solve the problem on hand. The success or failure of a population based algorithms depends on its ability to establish proper trade-off between exploration and exploitation. A poor balance between exploration and exploitation may result in a weak optimization method which may suffer from premature convergence, trapping in a local optima and stagnation. PSO algorithm is another example of population based algorithms (Ardito et al. 2005). PSO is a stochastic optimization technique which is well adapted to the optimization of nonlinear functions in multidimensional space and it has been applied to several real-world problems (Boehner et al. 2007; Khan and Sahai 2012).

The gravity method was the first geophysical technique to be used in oil and gas exploration. Despite being eclipsed by seismology, it has continued to be an important and sometimes crucial constraint in a number of exploration areas. In oil exploration the gravity method is particularly applicable in salt provinces, over thrust and foothills belts, underexplored basins, and targets of interest that underlie high-velocity zones. The gravity method is used frequently in mining applications to map subsurface geology and to directly calculate ore reserves for some massive sulfide ore-bodies. There is also a modest increase in the use of gravity techniques in specialized investigations for shallow targets. Also it has application in agriculture and archeology. Data reduction, filtering, and visualization, together with low-cost, powerful personal computers and color graphics, have transformed the interpretation of gravity data. Also in gravity methods, Euler and Werner deconvolution depth and edge -estimation techniques can help define the lateral location and depth of isolated faults and boundaries from gravity data. Complex geology with overlapping anomalies arising from different depths can, however, limit the effectiveness of deconvolution fault-detection results (Nabighian et al. 2005; Toushmalani 2010b; Toushmalani 2010c; Toushmalani 2010d; Toushmalani 2011).

The outline of this paper is as follows. In first section we discussed the gravity field of a fault, Section Levenberg-Marquardt describes the algorithms of PSO and LM. Section Particle Swarm Optimization (PSO) presents application of Levenberg-Marquardt backpropaction algorithm, and a particle swarm algorithm in solving inverse problem of a fault. Most importantly the parameters for the algorithms are given for the individual tests. Section Application of PSO and LM optimization in inverse problem solving presents conclusions and final comments.

### Appplication to the gravity field of a fault

**Figure 1:**
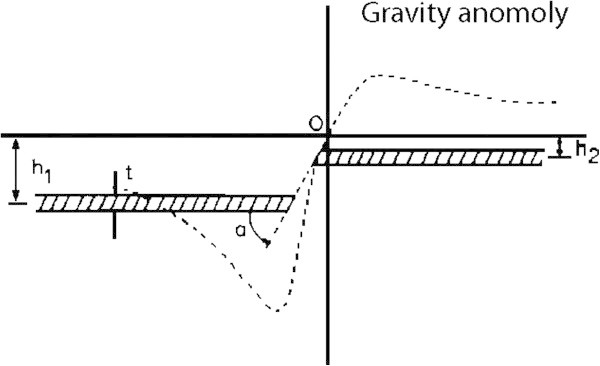
**Fault model illustrating various parameters used in work, and shape of expected gravity anomaly.**

A fault structure can be approximated by two Semi-infinite horizontal sheets, one displaced vertically from the other. The general situation of a fault is presented in Figure 1, together with the shape of the Expected anomaly which is described by the formula 2 (Telford et al. 1976):2

K: =6.672e-3;

б: =density contrast; t: =thickness of sheet; h_1,2_=depth of each side to the middle of the sheet; a: =fault angle (Thanassoulas et al. 1987; Telford et al. 1976; Toushmalani 2010a; Toushmalani 2013).

### Levenberg-Marquardt

**Figure 2:**
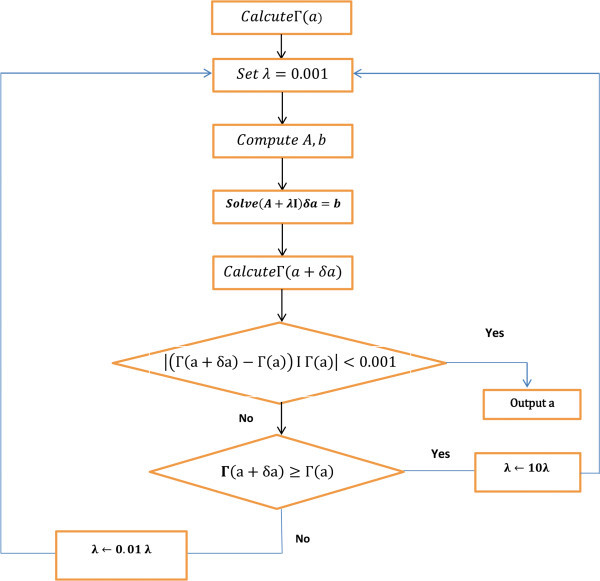
**Flow chart of Levenberg-Marquardt.**

The Levenberg-Marquardt algorithm (LM) (Juutinen and Saariluoma 2007) is an approximation to the Newton method (cf. Figure 2) used also for training ANNs.

The Newton method approximates the error of the network with a second order expression, which contrasts to the Backpropagation algorithm that does it with a first order expression. LM updates the ANN weights as follows:3

Where *J*^*p*^ (*w*) is the Jacobian matrix of the error vector *e*^*p*^ (*w*) evaluated in w, and I is the identity matrix. The vector error *e*^*p*^ (*w*) is the error of the network for pattern p, that is, *e*^*p*^(*w*) = *t*^*p*^ − *o*^*p*^(*w*).

**Figure 3:**
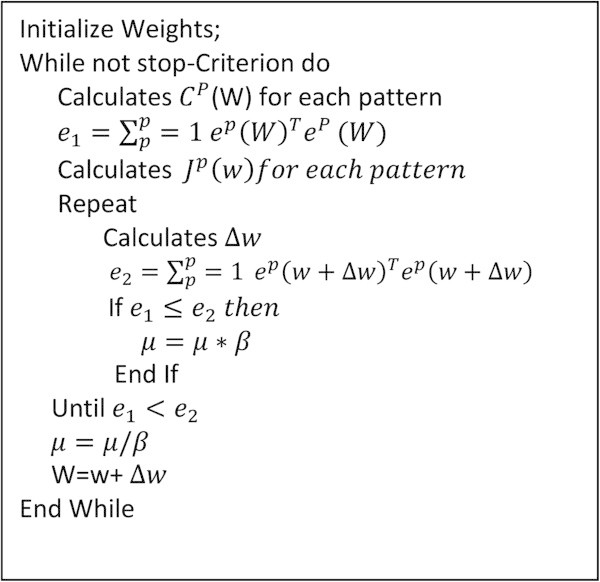
**Pseudocode of Levenberg-Marquardt algorithm.**

The parameter μ is increased or decreased at each step. If the error is reduced, then μ is divided by a factor *β*, and it is multiplied by *β* in other case. Levenberg -Marquardt performs the steps detailed in Figure 3. It calculates the network output, the error vectors, and the Jacobian matrix for each pattern. Then, it computes ∆w using (3) and recalculates the error with w + ∆w as network weights. If the error has decreased, μ is divided by *β*, the new weights are maintained, and the process starts again; otherwise, μ is multiplied by *β,* ∆w is calculated with a new value, and it iterates again (Khan and Sahai 2012).

### Particle swarm optimization (PSO)

**Figure 4:**
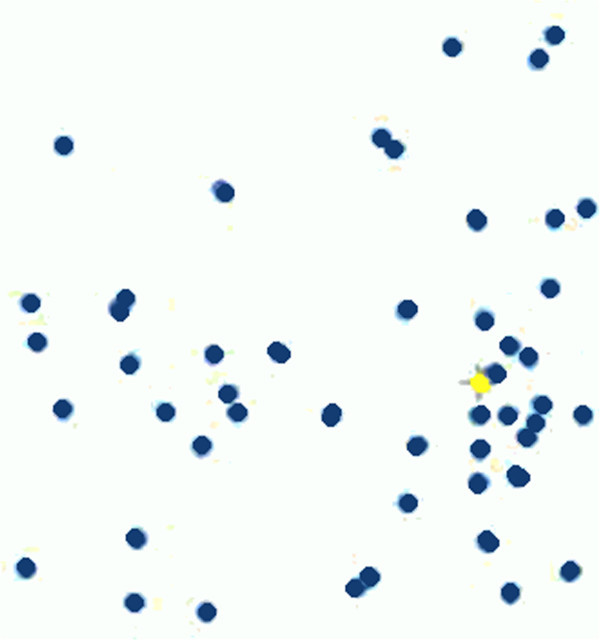
**Particles movement in PSO.**

The PSO algorithm was first introduced by Eberhart and Kennedy (Council of Ministers of Education 1998; ISO 9241 1998; Chiu et al. 2005; Hannula 2006). Instead of using evolutionary operators to manipulate the individuals, like in other evolutionary computational algorithms, each individual in PSO flies in the search space with a velocity which is dynamically adjusted according to its own flying experience and its companion s’ flying experience. Each individual is treated as a volume-less particle (a point) in the D-dimensional search space (cf. Figure 4) (Khan and Sahai 2012).

The ith particle is represented as *X*_*i*_ = (*x il*, *x i*2 , …, *x*_*iD*_). The best previous position (the position giving the best fitness value) of the ith particle is recorded and represented as *P*_*i*_ = (*p il*, *p i*2 , …, *p*_*iD*_). The index of best particle among all the particles in the population is represented by the symbol gb representing global best. The index of the best position for each particle in the population is represented by the symbol ib representing the individual’s best. The rate of the position change (velocity) for particle i is represented as *V*_*i*_ to the following equation: (*v*_*i*1_, *v*_*i*2_ , …, *v*_*iD*_). The particles are manipulated according to the following equations:4

5

The algorithm can be summarized as follows:

- Step 1. Initialize position and velocity of all the particles randomly in the *N* dimension space.
- Step 2. Evaluate the fitness value of each particle, and update the global optimum position.
- Step 3. According to changing of the gathering degree and the steady degree of particle swarm, determine whether all the particles are re-initialized or not.
- Step 4. Determine the individual best fitness value. Compare the *p*_*i*_ of every individual with its current fitness value. If the current fitness value is better, assign the current fitness value to *p*_*i*_.
- Step 5. Determine the current best fitness value in the entire population. If the current best fitness value is better than the *p*_*g*_, assign the current best fitness value to *p*_*g*_.
- Step 6. For each particle, update particle velocity,
- Step 7. Update particle position.
- Step 8. Repeat Step 2–7 until a stop criterion is satisfied or a predefined number of iterations are completed (Khan and Sahai 2012; Toushmalani 2013).

**Figure 5:**
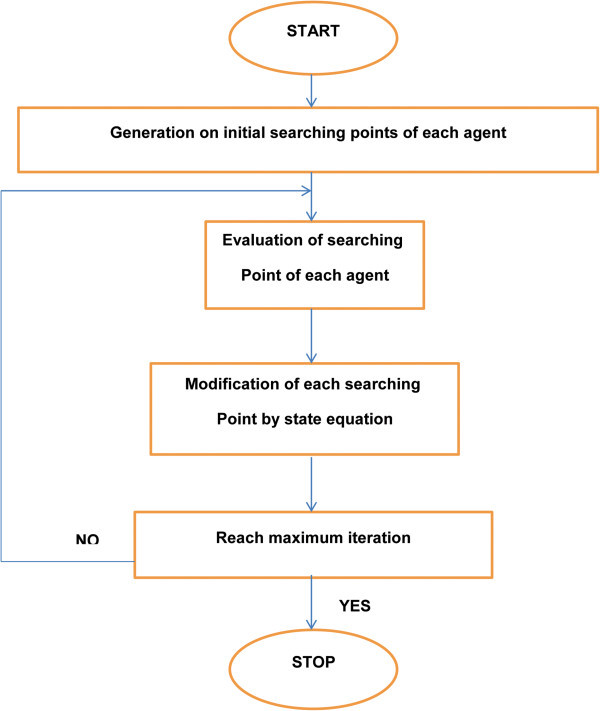
**PSO Flowchart.**

The particle swarm flowchart is shown on Figure 5.

### Application of PSO and LM optimization in inverse problem solving

Using Equation (1), the theoretical anomaly which corresponds to a fault with t = 500 m, h_1_ = 6000 m (left), h 2 = 2000 m, a = 30°, and б = 1, is presented as a continuous line in Figure 2. To test the program, the theoretical anomaly of Figure 2 is digitized every 5000 m (Table 1), and a “bad” initial model with parameters h1 = 3000 m, h2 = 1600 m, t = 700 m, and a = 30° is entered (Thanassoulas et al. 1987). Table 1 shows Gravity anomaly for inversion.

**Table 1:** **Gravity anomaly for inversion**

Gravity anomaly (mgal)	x-coordinate (m)
−2.24	−15000
−3.47	−10000
−5.60	−5000
0	0
2.02	5000
1.61	10000
1.27	15000
1.04	20000

During the iterations the density contrast is kept as a fixed parameter, assuming that its value has been estimated previously. The parameters which are optimized are:

- the thickness of the sheet,
- the left distance to the middle of the sheet,
- the right distance to the middle of the sheet, and
- the angle of the fault.

Table 2. Parameters of obtained solution with Levenberg-Marquardt (Thanassoulas et al. 1987).

 Thickness of fault: 500 m

 Fault angle (a): 60°

 Depth to bottom of the fault (h1): 5780 m

 Depth to top of the fault (h2): 1753 m

 -. Parameters of obtained solution with PSO:

 Thickness of fault: 501.44 849 m;

 Fault angle (a): 1.0 5*p - p =189-180 = 9°,

 Depth to bottom of the fault (h1): 6000 m;

 Depth to top of the fault (h2): 2001.6431 m; (Toushmalani 2013). Table 2 shows Parameters of obtained solution.

**Table 2:** **Parameters of obtained solution**

Calculated gravity with LM	Calculated gravity with PSO	Observed gravity
−2.23	−2.23	−2.24
−3.48	−3.47	−3.47
−5.84	−5.60	−5.60
0	0	0
2.15	2	2.02
1.67	1.63	1.61
1.30	1.29	1.27
1.06	1.05	1.04
2.5%	0.5%	RMS

The mean squared error function was used as the training error. The term root mean square error (RMSE) is the square root of mean squared error (MSE). RMSE measures the differences between values predicted by a hypothetical model and the observed values. In other words, it measures the quality of the fit between the actual data and the predicted model. RMSE is one of the most frequently used measures of the goodness of fit of generalized regression models.

## Conclusion

The parameters which are optimized with these methods are: (a) the thickness of the sheet, (b) the left distance to the middle of the sheet, (c) the right distance to the middle of the sheet, and (d) the angle of the fault. Inverse solution reveals that fault model parameters are agree quite well with the known results. A more agreement has been found between the predicted model anomaly and the observed gravity anomaly in PSO Method rather than LM method.
